# Molecular Cytogenetic Analysis in Freshwater Prawns of the Genus *Macrobrachium* (Crustacea: Decapoda: Palaemonidae)

**DOI:** 10.3390/ijms21072599

**Published:** 2020-04-09

**Authors:** Wagner F. Molina, Gideão W. W. F. Costa, Inailson M. C. Cunha, Luiz A. C. Bertollo, Tariq Ezaz, Thomas Liehr, Marcelo B. Cioffi

**Affiliations:** 1 Departamento de Biologia Celular e Genética, Centro de Biociências, Universidade Federal do Rio Grande do Norte, Natal, RN 59078970, Brazil; molinawf@yahoo.com.br (W.F.M.); wagnerwf@yahoo.com.br (G.W.W.F.C.); inailsonmarcio@gmail.com (I.M.C.C.); 2 Laboratório de Citogenética de Peixes, Departamento de Genética e Evolução, Universidade Federal de São Carlos, São Carlos, SP C.P. 676, Brazil; bertollo@ufscar.br (L.A.C.B.); mbcioffi@ufscar.br (M.B.C.); 3 Institute for Applied Ecology, University of Canberra, Canberra, ACT 2617, Australia; Tariq.Ezaz@canberra.edu.au; 4 Institute of Human Genetics, University Hospital Jena, 7747 Jena, Germany

**Keywords:** crustacea cytogenetics, karyotype evolution, rDNA, SSRs, diploid variation

## Abstract

Freshwater prawns of the genus *Macrobrachium* are one of the important components of circumtropical marine, estuarine, and freshwater environments. They have been extensively exploited for human consumption for many years. More than 250 species reflect the evolutionary success of this highly diversified group, with a complex and challenging taxonomy due to morphological variations and vast geographical distribution. Although genetic approaches have been used to clarify phylogenetic and taxonomic aspects of *Macrobrachium* species, cytogenetic information is still very scarce and mostly focused on chromosome number and morphology. Here, we present chromosome data for three species from the Neotropical region, *M. carcinus,* *M. acanthurus,* and *M. amazonicum*, and one species from the Oriental region, *M. rosenbergii*. Using conventional cytogenetic approaches and chromosome mapping of repetitive DNAs by fluorescence *in situ* hybridization (FISH), we identified numerical diversification of the diploid set, within and between both zoogeographic regions. These included *M. acanthurus* and *M. amazonicum* sharing diploid chromosomes of 98, while *M. carcinus* has 94, and *M. rosenbergii* has 118 chromosomes. Argentophilic sites are also variable in number, but they occur in a much higher number than 18S rDNA, representing two to 10 sites within the study species. Microsatellites repeat motifs are also abundant in the chromosomes, with a co-localization and uniform distribution along the chromosome arms, but completely absent in the AT-rich centromeric regions. As a whole, our study suggests that the 2n divergence was followed by a considerable rDNA diversification. The abundance of the exceptional amount of microsatellite sequences in the chromosomes also suggests that they are essential components of the *Macrobrachium* genome and, therefore, maintained as a shared feature by the species, the reason for which is yet unknown.

## 1. Introduction

Palaemonidae (Decapoda) is a diverse family of shrimp having considerable economic interest [1], comprising 137 genera and more than 950 species distributed in marine, estuarine, and freshwater environments around the world [2]. In this family, the genus *Macrobrachium* Spence Bate, 1868, constitutes a very diversified group comprising more than 250 species [3], and important links in trophic chains of lakes, rivers, and estuarine areas of tropical and subtropical regions [4,5]. The species of this genus are characterized by a second pair of very elongated locomotory appendices, generally equal to or larger than the body size, with prominent chelae [6].

This genus was probably originated during the later Cretaceous, in the Sea of Tethys, whose fragmentation promoted its disjunct distribution and consequent isolation of the American and African strains that are relics of this period [7]. The greatest diversity of species occurs in the Indo-Pacific region, including India and Southeast Asia, parts of Oceania and some Pacific islands, whereas other lineages are found along the west African coast [8], and in Americas, from the south of the USA to the south of Brazil [9,10,11].

*Macrobrachium* species are important resources in aquaculture and fisheries in many countries [12]. Among them, the giant river prawn, *M. rosenbergii*, has been the focus of scientific and economic attentions due to its intensive use in aquaculture worldwide [13]. Therefore, some karyotype and genome data are already available for this species [14,15,16,17]. However, native species from different countries have also been increasingly used for aquaculture purposes [18]. In turn, from the biological conservation view, *M. rosenbergii* represents a serious risk to ecosystems of many countries where it is invasive [19], including Brazilian regions comprising a large number of congeneric species [20,21,22]. 

The real diversity of *Macrobrachium* is still largely unknown, with continuous taxonomic reviews and descriptions of new species [23,24,25,26]. The extensive geographic distributions of some species, associated with highly variable morphological characters [27,28], have driven the integrated use of molecular and morphological data to elucidate their challenging taxonomy (e.g., [29,30]).

Although unexpected given its notable diversity (>8000 species) and economic importance, cytogenetic data are scarce in Decapoda, particularly among *Macrobrachium* species [31,32]. Low mitotic indices, high 2n numbers, small chromosomes, and technical limitations [33,34] are possible reasons for the restricted data on karyotype evolution in this group. Only a few dozen species of Decapoda have some cytogenetic information and, when available, mainly related to the diploid number or karyotype structure, with very few reports on molecular cytogenetics or chromosome banding [31,32]. This is also true for *Macrobrachium* genus, whose cytogenetic data encompass less than 5% of its diversity (Table 1). 

At least 55 *Macrobrachium* species are found in American regions, 17 of which inhabit Brazilian waters [30]. Therefore, accessing their cytogenetic data can provide important clues to understand evolutionary processes in the genus. The objective of present study is to use conventional cytogenetic approaches, as well as FISH mapping of rDNA and repetitive sequences to characterize chromosome organization and compare karyotype characteristics across four *Macrobrachium* species, three from Brazil (*Macrobrachium acanthurus*, *M. amazonicum*, *M carcinus*) and *M. rosenbergii* to infer karyotype evolution within this group.

## 2. Results

**Figure 1:**
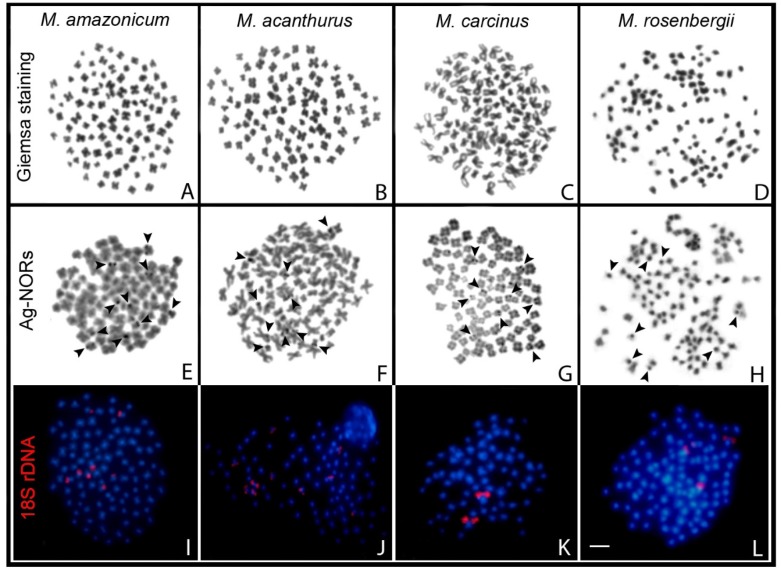
Spermatogonial metaphases of *Macrobrachium amazonicus* (**A**,**E**,**I**), *M. acanthurus* (**B**,**F**,**J**), *M, carcinus* (**C**,**G**,**K**), and *M. rosenbergii* (**D**,**H**,**L**) after Giemsa staining (**A**–**D**); Ag-NOR impregnation (**E**–**H**) and FISH with 18S rDNA (red) probe (**I**–**L**). Blue fluorescence represents chromosomes stained with DAPI and dark arrowheads highlight Ag^+^ signals in the chromosomes. Bar = 5 µm.

The four *Macrobrachium* species studied all possess small metacentric (m), submetacentric (sm), and subtelocentric (st) chromosomes, with the smallest pairs presenting <1.0 µm in size. The species of the Neotropical region have smaller diploid numbers in comparison with that from the Oriental region. Thus, *M. carcinus* possesses 2n = 94, and *M. acanthurus* and *M. amazonicum* 2n = 98, whereas *M. rosenbergii* has 2n = 118 chromosomes, which is consistent with previous descriptions for these species [14,15,42]. No evidence for male chromosomal heteromorphism was detected at the level of the present investigation (Figure 1).

Large DAPI^+^ centromeric heterochromatic blocks are present in all four species, which are amplified and reach the pericentromeric region in some chromosome pairs. Ag^+^ signals are generally present on the short arms of the chromosomes and, to a lesser extent, in the pericentromeric position, but with intra- and interindividual variation in number (Figure 1). 

FISH mapping of the 18S rDNA revealed different numbers of sites for this repetitive family, comprising eight sites in *M. amazonicum*, 10 in *M. acanthurus*, two in *M. carcinus*, and four sites in *M. rosenbergii*. In each species, the 18S rDNA sites occupy the terminal regions of chromosome pairs (Figure 1I–L). Noticeably in *M. carcinus* these sites were larger than in the other species. No specific correlation between the Ag^+^ and the 18S-5.8S-28S rDNA loci was observed, the former being much more frequent in the karyotype (Figure 1E–L). 

**Figure 2:**
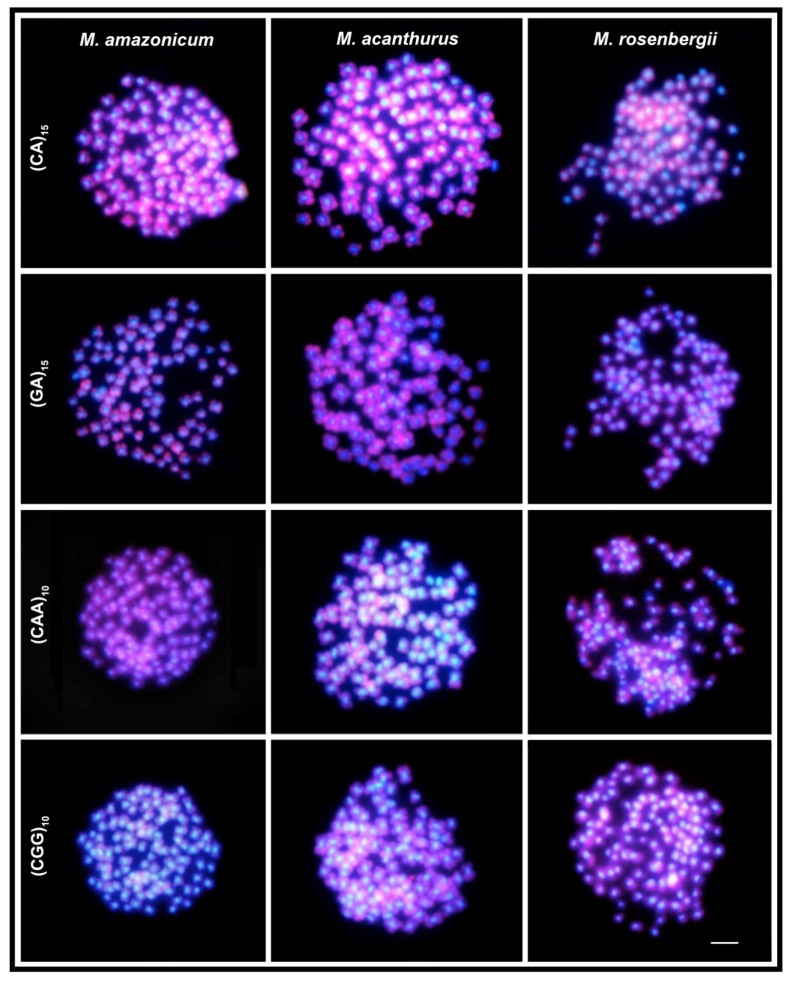
Fluorescence *in situ* hybridization with microsatellite probes (red fluorescence) in spermatogonial metaphases of *Macrobrachium* species. Blue fluorescence represents chromosomes stained with DAPI. Bar = 5 µm.

The mapping of the simple sequence repeats (SSRs) ((CA)_15_, (GA)_15_, (CAA)_10_, and (CGG)_10_) showed a surprisingly large amount of accumulation/amplification distributed homogeneously along the chromosome arms. However, these sequences are absent in the centromeric regions, where large AT-rich heterochromatic blocks (DAPI^+^) occur (Figure 2). No hybridization signal with the (TTAGGG)n probe was found in any species.

## 3. Discussion

Decapoda display some of the largest diploid chromosome numbers among animals, with variations ranging from around 50 to hundreds of chromosomes, as in the crab *Liocarcinus vernalis*, 2n = 54 [43] and the hermit crab *Pagurus ochotensis*, 2n = 254 [44,45]. Such variability is also found in the four species we have now investigated (2n = 94–118), and, including the published karyotype information, the diploid range for these species is now (2n = 94–124) (Table 1). According to [41], higher 2n values constitute an evolutionarily ancestral condition for Decapoda species, thus indicating a karyotype evolution mediated by a variety of chromosome rearrangements, with the Robertsonian translocation playing a prominent role. 

The variability of the chromosome numbers contrasts with what occurs in other Decapoda representatives, which exhibit a marked uniformity in their chromosomal numbers. In fact, some marine groups such as Penaeidae, with great dispersive potential [46,47], have more stable 2n numbers (e.g., [48,49]). In contrast, *Macrobrachium* (Palaemonidae), whose representatives are restricted to freshwater environments, or with dependence on saline environments and with reduced gene flow [50,51], presents a marked diversification. In this respect, ecological aspects deserve to be better understood concerning their probable role in karyotype diversification or stasis (*sensu* [52]).

**Figure 3:**
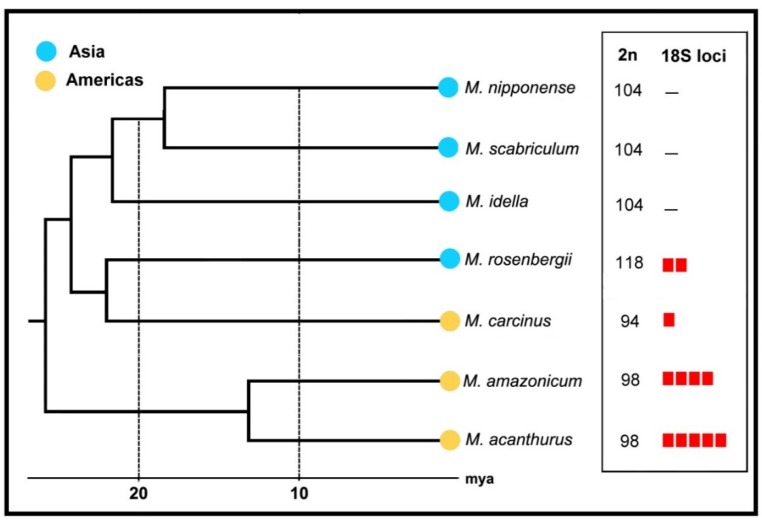
Diploid numbers and frequency of 18S-5.8S-28S rDNA loci in *Macrobrachium* species from a biogeographic and phylogenetic perspective. The red squares represent the number of chromosome pairs bearing these sequences (adapted from [55]).

Historical biogeography indicates that *Macrobrachium* species composition results not only from local divergences [53], but also includes long-distance dispersal of different phylogenetic lineages [54]. From a biogeographic context, *Macrobrachium* possesses contrasting patterns of chromosome numbers, with the American species showing comparatively lower 2n numbers (94–98) and the major Asian species with 2n = 100–118 (Table 1; Figure 3). The causes of this divergence are not known, although it is congruent with the exclusivity of clades between both continents. Interestingly, *M. carcinus* (from the Americas) has 2n = 94 and *M. rosenbergii* (from Asia) has 2n = 118. Although belonging to the same clade [55], their chromosomal numbers are linked with the karyotype trends in each biogeographic region rather than their common phylogenetic position. Noteworthily, this case highlights independent evolutionary conditions for phylogenetically close species. It is expected that the increase of cytogenetic research will allow us to better understand such differential numerical patterns of karyotypes between the American and Asian biogeographic regions.

Repetitive DNAs are very informative sequences about karyotype evolution and, among them, the 18S-5.8S-28S rRNA genes stand out as one of the most studied ones in the eukaryote genome [56]. The Ag-NOR staining procedure allows us to identify the 18S-5.8S-28S rRNA genes that were transcriptionally active during the previous interphase [57]. However, in *Macrobrachium* species, a very large number of Ag^+^ signals were found, which far exceed the number of 18S rDNA sites unveiled by FISH procedures. It is likely that this characteristic may be related to the argentophilic properties of centromeric proteins [58,59], or pseudo-NORs [60]. Pseudo-NORs are tandem sequences of heterologous DNA sequence with high affinity for a DNA binding protein (upstream binding factor), part of the Pol I transcription machinery that binds across the rDNA repeats [61]. These regions mimic active NORs in a number of important respects, among them the presence of acidic residues, with strong affinity by silver, that could provide additional Ag+ sites without relation with NORs.

In giant freshwater prawns, the 18S rDNA was proved to be a discriminating cytotaxonomic character, with a marked phylogenetic link. Accordingly, the number of loci is particular for each one of the analyzed species, with similarities for those phylogenetically close ones (Figure 3). While *M. acanthurus* and *M. amazonicum* have higher numbers of 18S rDNA sites (10 and eight, respectively), their significantly lower numbers are found in *M. carcinus*, *M. lanchesteri* [32], and *M. rosenbergii* (two, four, and four sites, respectively). The occurrence of more than one 18S rDNA sites has been reported in several Decapoda groups [31,59,62], suggesting that it may represent an ancestral condition for the order. If so, the single chromosome pair bearing 18S rDNA sequences in *M. carcinus* would indicate a reorganization of this repetitive family of sequences in the karyotype of this species. 

Telomeres are highly repeated DNA sequences that protect the ends of the chromosomes [63] and show striking similarity even in evolutionarily distant organisms [64]. In several crustacean groups discernible telomeric signals have been reached using as (TTAGGG)n as (TTAGG)n probes [65,66]. However, in *Macrobrachium* species the FISH experiments with (TTAGGG)n probes did not produce detectable signals. Therefore, further investigations are needed to improve the hybridization method and understand the role of telomeric sequences in the karyotype evolution of *Macrobrachium*. Another striking characteristic in *Macrobrachium* species is their large content of AT-rich segments in the centromeric regions. However, this pattern has a broader phylogenetic extension, including other species of the genus [34], other Palaemonidae genera [31], and Decapoda families [66], thus indicating a stable condition for the order.

The variation of DNA content is also remarkable in Palaemonidae, comprising 5.0× at the family level, and 3.4× particularly among *Macrobrachium* species. The causes of this variation are not entirely understood in crustaceans [67]; however, the participation of retrotransposons has been identified as one of the main factors in the expansion of the genome in the family [68] and deserves to be clarified using more extensive cytogenetic and genomic approaches. Repetitive sequences, including transposable elements (TE) and microsatellite DNAs, occupy a considerable portion of some Crustacean genomes [69]. TEs contribute to genome expansion and alteration not only by transposition but also by generating tandem repeats [70]. Associations among transposable elements and microsatellite expansions can play an important role in chromosome differentiation [71]. In this sense, estimative of the microsatellites content and organization in the *Macrobrachium* chromosomes open perspectives for future investigations about the evolutionary interrelationships of these repetitive DNA classes. In *Macrobrachium* species the mapping of four microsatellite classes revealed their striking content in the genome of species. Significantly, these SSRs overlap all chromosome arms but are absent in the centromeric AT-rich regions. This compartmentalized organization indicates that repetitive DNAs follow divergent and complex evolutionary paths in *Macrobrachium* species, besides appearing to be integrated with transcriptional regions of the genome.

Increasing invasions of *Macrobrachium* species occur worldwide as a result of their use in aquaculture [19]. Such events promote contact among geographically isolated species, raising several ecological and evolutionary questions. In South America, for example, the Asian *M. rosenbergii* has established invasive populations in areas where the native *M. amazonicum* occurs [21], a condition that also occurs in many other countries [22]. Therefore, it is increasingly necessary to evaluate the potential for intercrossing of *Macrobrachium* species, as a valuable parameter for biological conservation in the light of genetic introgression risks. Several pieces of evidence have highlighted the substantial interference of the hybridization on evolutionary processes [72,73]. However, its occurrence and effects are yet to be fully known for the freshwater giant prawns. 

Remarkably, hybridization experiments among phylogenetically close species from the same biogeographic region have resulted in viable *Macrobrachium* hybrids [74,75,76,77]. In contrast, induced crosses among phylogenetically closer species, but from distant biogeographic regions, produce zygotes which did not go beyond the gastrula stage, i.e., 4–6 days after fertilization [78]. Thus, in *Macrobrachium*, as in other organisms [79,80,81], post-zygotic reproductive barriers may result in unequal chromosomal segregations from crosses of individuals bearing different 2n numbers. Post-zygotic barriers probably related to differences in the chromosome number and distinct biogeographic areas have been reported in experimental crosses among several *Macrobrachium* species [82,83,84]. Thus, it is likely that the diversification of the diploid number and structural features reduce the success of hybridizations among natural and invasive species from different biogeographic regions. This condition is consistent with the long period of divergence that such lineages have experienced.

### Final Remarks

Conventional and molecular cytogenetic data are useful in estimating evolutionary patterns, as well as probable crossbreeding chances among *Macrobrachium* species. Apomorphic cytogenetic characteristics (2n numbers, karyotype formulas, and frequency and distribution of rDNA), in addition to other shared features (e.g., prevalence of two-armed chromosomes, organization and composition of specific heterochromatin), interact in the evolutionary process of this prawn group. Noteworthy, despite long periods of isolation in different biogeographic regions, some chromosomal characteristics remain conserved, suggesting that they play important roles in the evolutionary history of the genus. In this context, it stands out the striking amount and particular distribution of microsatellite sequences in the in nuclear genomes of analyzed species. Faced with a growing number of biological invasions, hybridization risks among *Macrobrachium* species are real and viable hybrids have been reported, with serious implications for worldwide aquatic ecosystems. Fortunately, divergences in the number of chromosomes allow us to estimate effective post-zygotic barriers. In this sense, investigations of such simple and useful cytogenetic indicators deserve to be increased given their important contribution towards genetic conservation.

## 4. Materials and Methods 

### 4.1. Species Analyzed

*Macrobrachium* males of three South American species, *M. carcinus* (Linnaeus, 1758), *M. acanthurus* (Wiegmann, 1836), and *M. amazonicum* (Heller, 1862), and one Asian species, *M. rosenbergii* (de Man, 1879) were analyzed. Natural samples of *M. carcinus* and *M. acanthurus* were from Jiqui Lake (5°55′07.7′′ S/35°11′17.6′′ W), municipality of Parnamirim. *M. amazonicum* was from an invasive population in the Seridó River (6°27′14.9′′ S/37°06′25.6′′ W), municipality of Caicó. Both locations are situated in the Rio Grande do Norte State, northeastern Brazil. Individuals of *M. rosenbergii* were obtained from a freshwater prawn farm in the Paraíba State, also in NE Brazil.

### 4.2. Chromosome Preparations 

Chromosomes were obtained from male gonads using the method described by Klingerman and Bloom [85], with modifications suggested by Lakra et al. [42]. The specimens received an injection of colchicine solution (2.0 µg/g of body weight) at the base of the third pair of pereiopods and were kept in constantly aerated aquariums for 6 h. The testicles were removed, sectioned into 1-mm pieces, and subjected to a 0.075 M KCl hypotonic solution for 40 min. After that, the fragments were fixed in a methanol plus acetic acid (3:1) solution, three changes 15 min each, and stored at –20 °C. Spermatogonial metaphases were obtained by macerating the tissues in 1.0 mL of 50% acetic acid solution with tweezers. About 150 µL of the cell suspension was dropped and immediately re-aspirated with a Pasteur pipette on heated slides at 55 °C and air-dried. Chromosomes were stained with 10% Giemsa solution diluted in phosphate buffer (pH 6.8), and analyzed under an optical microscope at 1200× magnification. Chromosomes were classified as metacentric (m), submetacentric (sm), subtelocentric (st), or acrocentric (a), according to their centromere positions [86].

The detection of the nucleolus organizing regions (Ag-NORs) was performed using the Silver nitrate staining procedure [87]. For fluorochrome staining, the slides were mounted with 30 µL of Vectashield anti-fading medium (Vector Laboratories, Burlingame, CA, USA) plus 1.0 µL/mL of DAPI, covered with coverslips and kept at 4 °C in the dark. The analyses were carried out using an Olympus™ BX51 epifluorescence photomicroscope with appropriate filters. The best results were photographed using the Olympus DP73 digital capture system with cellSens 1.7 (Olympus Soft Imaging Solutions, Münster, Germany). 

The experimental work fulfills all ethical guidelines regarding the handling of specimens. The collection and handling of specimens followed protocols approved by the Ethics Committee on the Use of Animals of the Federal University of Rio Grande do Norte (#044/2015).

### 4.3. Fluorescence In Situ Hybridization (FISH)

The 18S rDNA probes (1200 bp) were obtained by a polymerase chain reaction, from the nuclear DNA of *Macrobrachium carcinus* (Crustacea, Palaemonidae) using the NS1 5′-GTA GTC ATA TGC TTG TCT C-3′ and NS8 5′-TCC GCA GGT TCA CCT ACG GA-3′ primers [88]. Subsequently, the probe was labeled by nick translation with digoxigenin-11-dUTP (Roche, Mannheim, Germany). FISH experiments were performed according to [89]. Slides with chromosome preparations were washed with 1×PBS buffer for 5 min at 25 °C and subsequently dehydrated in an alcoholic series (70%/80%/100%). Next, the chromosomes were treated with DNAse-free RNAse (20 mg/mL in 2×SSC) at 37 °C for 1 h, with pepsin (0.005% in 10 mM HCl) at 37 °C for 10 min, fixed with 1% formaldehyde for 10 min, and then dehydrated in an alcoholic series. The chromosomes were then denatured in 70% formamide/2×SSC at 72 °C for 5 min. A hybridization solution comprising 50% formamide, 2×SSC, 10% dextran sulfate, and the denatured probe (5 ng/µL) was applied on the slides overnight at 37 °C. After hybridization, the slides were washed in 15% formamide/0.2×SSC at 42 °C for 20 min, 0.1×SSC at 60 °C for 15 min, and 0.5% Tween20/4×SSC at room temperature. The hybridization signals were detected using anti-digoxigenin rhodamine (Roche, Mannheim, Germany). 

Oligonucleotides rich in microsatellite sequences [d(CA)_15_, d(GA)_15_, d(CAA)_10_, and d(CGG)_10_] were used as probes as described by [90] and labeled with Alexa-Fluor 555 (Invitrogen, Thermo Fisher Scientific, Waltham, MA, USA) directly during their synthesis (VCB-Biotech). Hybridizations with telomeric sequences (TTAGGG)n were performed using the Telomere PNA FISH/FITC kit (Dako, Glostrup, Denmark) following the manufacturer’s instructions. After FISH procedures, the chromosomes were counterstained with Vectashield/DAPI (1.5 µg/mL) anti-fade medium (Vector Laboratories, Burlingame, CA, USA) and analyzed in an epifluorescence microscope Olympus BX51 (Olympus Corporation, Ishikawa, Japan).

## Figures and Tables

**Table 1:** Cytogenetic data for the family Palaemonidae (Decapoda).

Species	2n	Karyotype	References
*Macrobrachium villosimanus*	124	22m+22sm+ 80t/st	[34]
*M rosenbergii*	118	52m+54sm+12st/t	[14]
*M. rosenbergii*	118	90m/sm+28st/t	[15]
*M. rosenbergii*	118	118 m/sm/st	Present study
*M. lamarrei*	118	8m+110t	[35]
*M. lanchesteri*	116	54m+46sm+10a+6t	[32]
*M. nipponense*	104	74m+22t+8st	[36]
*M. idella*	104	50m+24t+30a	[37]
*M. scabriculum*	104	22m+10t+22a+XY	[37]
*M. siwalikensis*	100	100m	[38]
*M. superbum*	100	60m+12sm+28t/st	[39]
*M. amazonicum*	98	98m/sm/st	Present study
*M. acanthurus*	98	98m/sm/st	Present study
*M. carcinus*	94	94m/sm/st	Present study
*Palaemon khori*	96	52m+14sm+24st+6t	[1]
*P. modestus*	90	56m+8sm+12st+14t	[40]
*P. elegans*	90/89	84/85m/sm+6/4a (X_1_X_2_Y)	[41]
*P. serratus*	56	4m+12sm+40t	[31]

Where m, metacentric; sm, submetacentric; st, subtelocentric; t, telocentric; and a, acrocentric chromosomes.
